# The Endocannabinoid System in the Postimplantation Period: A Role during Decidualization and Placentation

**DOI:** 10.1155/2013/510540

**Published:** 2013-10-21

**Authors:** B. M. Fonseca, G. Correia-da-Silva, M. Almada, M. A. Costa, N. A. Teixeira

**Affiliations:** ^1^Biologia da Inflamação e Reprodução, Instituto de Biologia Molecular e Celular (IBMC), Rua do Campo Alegre No. 823, 4150-180 Porto, Portugal; ^2^Laboratório de Bioquímica, Departamento Ciências Biológicas, Faculdade de Farmácia da Universidade do Porto, Ciências Biológicas Rua de Jorge Viterbo Ferreira No. 228, 4050-313 Porto, Portugal

## Abstract

Although the detrimental effects of cannabis consumption during gestation are known for years, the vast majority of studies established a link between cannabis consumption and foetal development. The complex maternal-foetal interrelationships within the placental bed are essential for normal pregnancy, and decidua definitively contributes to the success of this process. Nevertheless, the molecular signalling network that coordinates strategies for successful decidualization and placentation are not well understood. The discovery of the endocannabinoid system highlighted new signalling mediators in various physiological processes, including reproduction. It is known that endocannabinoids present regulatory functions during blastocyst development, oviductal transport, and implantation. In addition, all the endocannabinoid machinery was found to be expressed in decidual and placental tissues. Additionally, endocannabinoid's plasmatic levels were found to fluctuate during normal gestation and to induce decidual cell death and disturb normal placental development. Moreover, aberrant endocannabinoid signalling during the period of placental development has been associated with pregnancy disorders. It indicates the existence of a possible regulatory role for these molecules during decidualization and placentation processes, which are known to be particularly vulnerable. In this review, the influence of the endocannabinoid system in these critical processes is explored and discussed.

## 1. Cannabinoids: Historical Perspective

*Cannabis sativa* properties were known for centuries, though only in 1964 its main psychoactive component, Δ^9^-tetrahydrocannabinol (THC), was isolated and its chemical structure revealed. Due to its lipophilic nature, it was assumed that the psychotropic effects of THC resulted from interference with membrane fluidity, rather than binding to a specific receptor. However, by the mid 1980s, it was shown that cannabinoid activity was highly stereoselective, which led to the search for a specific receptor and its endogenous ligands [1, 2].

In late 1980s, cannabinoid receptors were discovered. The first cannabinoid receptor (CB1) was isolated from rat brain, [3] and, in 1993, a second receptor (CB2) was cloned from human promyelocytic leukaemia HL-60 cells [4].

Both cannabinoid receptors are G protein-coupled receptors (GPCRs), and their activation reduces adenylyl cyclase activity, leading to diminished cyclic adenosine monophosphate (cAMP) levels [5, 6]. Additionally, both receptors are coupled with intracellular signalling pathways related to activation of mitogen-activated protein kinases (MAPK). The CB1 is also coupled to ionic channels, inhibiting N- and P/Q-type voltage-gated calcium channels, activating A-type voltage-gated calcium channels, and inwardly rectifying potassium channels [5–7]. Furthermore, cannabinoids can modulate sphingolipid-metabolizing pathways by increasing intracellular levels of ceramide, an ubiquitous lipid second messenger [8].

On the other hand, as cannabinoids induced contractility of vascular smooth muscles independently of CB1 or CB2 receptors activation, it was suggested that other cannabinoid-like receptors may exist [9, 10]. Later, the orphan receptor GPR55 was suggested to be involved in non-CB1, non-CB2-mediated actions of cannabinoids [11]. Though with limited sequence homology with CB1 (13%) and CB2 (14%), GPR55 was suggested as a new cannabinoid receptor, the CB3 [12].

## 2. Endocannabinoid System

Besides THC, other molecules have been described to bind and activate cannabinoid receptors [13]. Some of these molecules were found to be produced by the organism and derived from arachidonic acid (AA), thus resulting in a new class of cannabinoids—the endocannabinoids (eCBs).

The first endocannabinoid, *N*-arachidonoylethanolamine, later called anandamide (AEA), was isolated in 1992 from pig brain by Raphael Mechoulam's group [14]. Three years later a second compound, the 2-arachidonoylglycerol (2-AG), was identified [15, 16].

Although cannabinoid receptors constitute the main targets of AEA, this molecule is capable of interacting with other molecular targets, such as the transient receptor potential vanilloid 1 (TRPV1) [17] and the peroxisome proliferator-activated receptors (PPARs) family [18, 19]. In opposition, 2-AG has higher affinity to CB1 and CB2 receptors than AEA, though it does not activate TRPV1.

Although AEA and 2-AG remain the best studied, other endogenous compounds may also bind cannabinoid receptors such as 2-arachidonoylglycerol ether (noladin ether, 2-AGE) [20], O-arachidonoylethanolamine (virodhamine) [21], *N*-arachidonoyl dopamine (NADA) [22], *N*-arachidonoyl glycine (NAGly) [23], and *Cis-*9,10-octadecanamide (oleamide or ODA) [24]. 

Like these molecules, other lipid mediators share endocannabinoid metabolic pathways. Although they are not able to bind to any of the cannabinoid receptors identified so far, these lipid messengers may influence endocannabinoid metabolism and function. These include the *N-*acylethanolamide family, particularly *N*-palmitoylethanolamide (PEA; C16:0), stearoylethanolamide (SEA, C18:0), and *N*-oleoylethanolamide (OEA; C18:1) [25].

Together with cannabinoid receptors and the endogenous compounds, the endocannabinoid system is also constituted by the putative membrane transporter and the enzymes responsible for the biosynthesis and degradation of endocannabinoids [26].

It is an accepted idea that endocannabinoids are released “*on demand,*” which means they are only produced when they are needed and on locals required. Based on the presence of intracellular AEA binding proteins, recent studies have been trying to prove the existence of AEA storage sites, believed to be adiposomes [27]. This hypothesis refutes the current conviction of an “*on demand*” production, so it must be carefully and extensively analysed.

The major endocannabinoids have different biosynthetic pathways, though both result from membrane precursors through enhanced intracellular Ca^2+^ concentrations. While AEA is synthesized from its precursor, the *N*-arachidonoyl-phosphatidylethanolamine (NAPE), by a specific phospholipase D (NAPE-PLD) [28], 2-AG is produced through a phospholipase C (PLC), producing 1,2-diacylglycerol (DAG), which may be, subsequently, converted to 2-AG by diacylglycerol lipase (DAGL) [29, 30].

Once synthesized, endocannabinoids are released to extracellular environment to target cannabinoid receptors, located in cell membranes, though AEA may also act on intracellular sites, such as TRPV1 receptor and T-type Ca^2+^ channels [31, 32]. Endocannabinoids appear to be inactivated through a two-step process involving the transport across the membrane, followed by two specific hydrolytic systems. Anandamide is primarily degraded by FAAH through hydrolysis into arachidonic acid and ethanolamine [33, 34]. Although FAAH can also degrade 2-AG [35] into glycerol and arachidonic acid, the main enzyme responsible for the inactivation of this compound is monoacyl glycerol lipase (MAGL) [36]. As AEA and 2-AG present structural similarities with polyunsaturated fatty acids, they can also serve as substrate for the inducible cyclooxygenase-2 (COX-2) and various lipoxygenases (LOXs) [37].

The current evidence indicates endocannabinoids as relevant modulators of several physiological functions not only in the central and autonomic nervous system but also in immune system, endocrine network, gastrointestinal tract, and in reproductive system [38].

During the last decade, the role of endocannabinoid system network in female reproduction has attracted major attention. Various evidences indicate a role for endocannabinoid elements during the preimplantation period. Endocannabinoids and both cannabinoid receptors have been described from the earliest stages of embryonic development to be involved in the regulation of blastocyst maturation, oviductal transport, implantation, and pregnancy maintenance. CB1 receptor is expressed in the embryo, in much higher levels than those in the brain [39]. Consistently, AEA was also found in much higher levels in mice nonpregnant uterus than in brain, which together with the changing levels of AEA with pregnancy status was indicative of a possible role for this lipid in early pregnancy events [40]. 

Endocannabinoid levels contribute to create the appropriate environment conducive to preimplantation embryo transport through the oviduct [41]. In fact, there is a regional regulation with higher expression of FAAH and NAPE-PLD in the ampulla and isthmus, respectively. This differential expression creates the appropriate AEA levels during oviductal transport. 

A similar phenomenon is observed in mice uterus during implantation where expression of AEA-metabolizing enzymes in mouse uterus is critical to define their concentration in implantation sites and consequently in the implantation outcome. In fact, just before embryo implantation, AEA declines to barely detectable levels at the site of implantation, and this change is believed to contribute to the receptive uterine state [42]. AEA can also induce differential signals in blastocyst differentiation and outgrowth. At low levels, cultured blastocysts exhibited accelerated trophoblast differentiation and outgrowth, while higher levels induce opposite effects [43, 44]. Studies regarding the underlying mechanism of these biphasic effects revealed that stimulatory and inhibitory effects on blastocyst function and implantation depend on different signal transduction pathways. While AEA at low doses activates ERK signalling pathway, at high concentrations it inhibits Ca^2+^ influx. Both effects occur through CB1 receptor [44]. The AEA-biphasic effects reveal AEA as a potential “cannabinoid sensor” mechanism, influencing crucial steps during early pregnancy. Nowadays, it is well accepted that the embryo is a target for natural and endogenous cannabinoids, raising the significance of cannabinoid signalling in female fertility.

Whilst endocannabinoid signalling is clearly critical in early pregnancy events, its effects during decidualization and placentation period and implications in pregnancy outcome remain largely undefined. 

## 3. Endocannabinoid System during Decidualization

Essential changes must occur in human endometrium to allow the establishment of pregnancy. These changes occur in the uterine endometrial stromal cells, which undergo a characteristic decidual cell reaction. Decidualization prepares the uterus for the trophoblast invasion that occurs during pregnancy. 

In human, decidualization is present in normal menstrual cycle during the late secretory phase [45], whereas in rodents decidualization is only a blastocyst-dependent process in normal pregnancy [46]. At the site of blastocyst attachment, the endometrial stromal cells undergo decidual reaction, in which stromal cells proliferate and differentiate into decidual cells [47]. Morphologically, this process involves the differentiation of elongated fibroblast-like cells into enlarged polygonal epithelial-like decidual cells. Human decidual cells produce specific molecules such as inflammatory mediators like IL-1, IL-6, IL-8, and TNF-*α* [48], various regulatory factors including relaxin, renin, prolactin (PRL), and insulin-like growth factor binding protein-1 (IGFBP-1), [45, 49] and specific extracellular matrix proteins, such as laminin, type IV collagen, and fibronectin [50].

Anomalies on decidual process predispose to pregnancy complications, including miscarriage, preeclampsia, foetal growth restriction, and preterm labour.

The rat, just like human, exhibits a highly invasive type of placental development with subsequent remodelling of the uterine tissues, being a suitable model for studying the mechanisms of decidualization [46].

**Figure 1:**
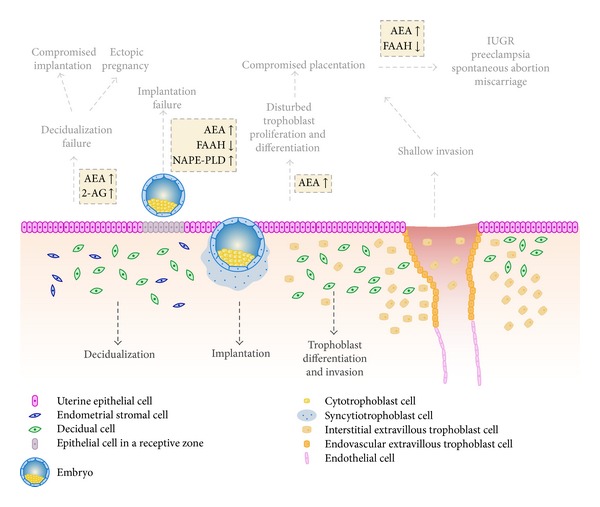
Schematic representation of the fetomaternal interface and potential adverse effects arising from deregulated endocannabinoid signalling based on rodents and human studies. Endometrial stromal cells differentiate into decidual cells, preparing uterine tissues for pregnancy, whereas the invading trophoblast cells critically regulate placental growth and function. All the physiological and molecular processes occurring during those periods are complex but highly organized. Endocannabinoids have reported to be involved in decidualization, implantation, and trophoblast differentiation and invasion. Aberrant endocannabinoid signalling (shown in yellow boxes) is reflected in compromised reprogramming of the endometrial stromal cells, implantation and placentation manifesting in ectopic pregnancy, intrauterine growth restriction, preeclampsia, miscarriage, and spontaneous abortion.

Studies in various mammals, including rats and humans, indicate that endocannabinoid system elements are present in decidua, which suggests its involvement in decidua establishment and/or remodelling (Figure 1) [51–55]. 

Although limited data are available concerning human decidual tissue, *Cb1* mRNA levels were detected in decidua from women with viable pregnancies [56–58], as well as immunoreactivity for CB1, CB2, NAPE-PLD, and FAAH proteins [55]. During the follicular phase of menstrual cycle, AEA plasmatic levels were significantly higher than those in the luteal phase [59], suggesting that steroid hormones may also be involved in the regulation of AEA levels in human pregnancy as previously observed during early pregnancy in mice [60]. Together, these data point to a full functional endocannabinoid system naturally occurring in human decidual tissue during pregnancy. Currently, there are no studies considering the expression of 2-AG metabolic enzymes or 2-AG levels during human pregnancy. 

In rodents, the stimulus for decidualization is not spontaneous, being the blastocyst crucial for this process. Detectable levels of proteins and respective mRNAs for metabolic enzymes (*Faah*, *Nape-pld*, Cox-2, *Magl*, and *Dagl*α**) and cannabinoid receptors (*Cb1, Cb2, Gpr55,* and *Trpv1*) in rat decidua throughout pregnancy [51–54] were found. Among these, CB1 was markedly upregulated during midpregnancy, which corresponds in rodents to the maximum decidua development with subsequent regression to allow placental establishment [51]. 

Additionally, it was observed that FAAH, but not NAPE-PLD activity, varies significantly throughout pregnancy in rat maternal tissues. In fact, there is an increase in FAAH activity once decidua is fully developed, suggesting that a tight regulation of AEA levels is required during maternal tissues remodelling and supports a successful pregnancy (unpublished data).

The major endocannabinoids, AEA and 2-AG, and the endocannabinoid-like compounds, OEA and PEA, are detected in rat plasma and decidua during the postimplantation period [52, 53]. Contrary to AEA, in which plasmatic levels were increased on day 10, the other analysed compounds (2-AG, OEA, and PEA) remained relatively unchanged during the postimplantation period [52, 53]. However, the tissue levels for all the studied EC fluctuate according to the period of pregnancy. Collectively, the tissue levels indicate that all the studied compounds may be required during normal pregnancy. However, the levels of these molecules in plasma do not reflect the concentrations in uterine tissues, suggesting that they are tissues regulated [52, 53].

Unlike AEA and 2-AG, OEA and PEA are not able to activate CB1 and/or CB2 receptors or modulate cell survival and death [61, 62]. However, they may potentiate endocannabinoid biological actions through interference with their degradation, a so-called “entourage” effect, thereby leading to an enhancement of EC effects [63, 64]. In that way, their levels also need to be tightly regulated otherwise, they could exacerbate AEA actions and consequently impair normal pregnancy.

Besides a full endocannabinoid system present in decidual cells, a functional effect occurring during decidualization as result of CB1 activation was observed. Kesser et al. evidenced that WIN, a synthetic cannabinoid, inhibits the induction of human decidual cell differentiation, by decreasing mRNA levels of various decidualization-specific markers like prolactin, laminin, and IGFBP-1 [56]. Indeed, WIN-exposed cells showed a marked reduction in intracellular cAMP levels causing important changes in the morphology of decidual fibroblasts with DNA fragmentation. All these effects were reversed by the CB1 antagonist indicating that activation of CB1 inhibits human decidualization and stimulates apoptosis by a cAMP-dependent mechanism [56].

During the past few years, endocannabinoid effects have been extensively studied in several cell types, and, particularly for AEA, a proapoptotic effect has been demonstrated in endothelial cells [65], human neuroblastoma CHP100, and lymphoma U937 cells [66]. However, contrary effects have also been observed, like protecting cells from apoptosis [67] or stimulating proliferation of cancer cells [68]. 

Concerning decidual cells, AEA and 2-AG were described as proapoptotic compounds in primary rat decidual cells [52, 69]. While lower concentrations induced morphologic and molecular alterations, characteristic of an apoptotic cell death, higher concentrations resulted in a dramatic effect on cell viability and morphology and an increase in LDH release, probably due to a necrotic effect [52, 69]. This suggests a dual effect for endocannabinoids during fetoplacental development, which is dependent on endocannabinoid concentration.

**Figure 2:**
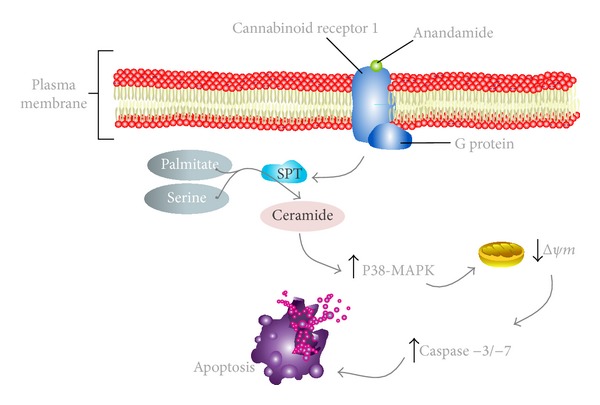
Schematic cartoon portraying the apoptotic signalling pathway triggered by anandamide (AEA) in rat decidual cells. AEA binds and activates the specific G-protein-coupled cannabinoid receptor 1 (CB1). The CB1 activation results in intracellular ceramide accumulation through *de novo* synthesis. This would lead to subsequent increase in phosphorylation of p38 mitogen-activated protein kinase cascade (p38-MAPK), which thus affects the mitochondrial pathway. It is followed by a drop in mitochondrial membrane potential (Δ*ψm*), caspase-3/-7 activation, and apoptosis of decidual cells. This CB1 activation mechanism is believed to play a role in decidual cell death, thus affecting uterine remodeling processes occurring during placentation.

On the other hand, the blockage of CB1 receptor, but not CB2 or TRPV1, was able to reverse the reduction of cell viability and apoptotic features induced by the two main endocannabinoids. Also, the activation of CB1 results in ceramide synthesis *de novo* and p38 phosphorylation, followed by induction of mitochondrial stress and ROS production, leading to apoptosis (Figure 2) [70]. Moreover, methyl-*β*-cyclodextrin (MCD), a cholesterol membrane depletor, has no effects on AEA/2-AG-programmed cell death [52, 69]. However, it has been referred that MCD blocks AEA-induced apoptosis in glioma cells [71] and hepatocytes [72]. This may result from CB1 redistribution in result of lipid raft disruption, as shown for breast cancer cells [73]. Furthermore, pretreatment with MCD increased decidual cell viability and caused a considerable reduction in LDH release only in the case of high concentrations of AEA and 2-AG [52, 69]. Thus, it is reasonable to suggest that high levels of AEA/2-AG, due to their lipophilic nature, may exert direct effects on rat decidual cells due to greater access through cholesterol-rich lipid rafts or through a membrane transporter present in these cells. Once inside the cell, these molecules induce detrimental effects that result in high cell cytotoxicity. In that way, depletion of membrane cholesterol inhibits this process and consequently inhibits cytotoxic effects without affecting the CB1-mediated apoptosis observed with the lower concentrations.

This evidence clearly indicates that membrane lipid composition and integrity may affect endocannabinoid signalling and uptake as previously observed in hepatic stellate cells. In these cells, alterations of membrane structure and cholesterol content reversed the cytotoxic effect of AEA/2-AG induced via mitochondrial reactive species [74, 75]. Consistently, CB1 activation in trophoblast cells during implantation may trigger different signalling pathways dependent on AEA levels [44].

Consistently with all these observations, an association between endocannabinoid system and decidua-related pregnancy disorders was shown. Lower CB1 expression was observed in decidua and fallopian tubes of women with ectopic pregnancy [76]. Additionally, AEA, OEA, and PEA plasmatic levels were all found to be significantly higher, whereas FAAH activity, but not NAPE-PLD activity, was significantly reduced in ectopic pregnancy [77]. These data suggest that aberrant endocannabinoid signalling in human decidua may result in ectopic pregnancy. Moreover, it points to a potential association between CB1 gene polymorphism and ectopic pregnancy.

Furthermore, AEA induces an increase in nitric oxide (NO) synthesis on decidua, which may implicate endocannabinoids in pathological reproductive events involving infection. These effects were abrogated by either co-incubation with CB1 or CB2 antagonists which suggests that both receptors could be mediating this effect [78].

Interestingly, it was observed that ECS regulates migration of endometrial stromal cell. More precisely, the synthetic cannabinoid methanandamide enhanced endometrial stromal cells migration via CB1, through the activation of PI3K/Akt and ERK1/2 pathways [79]. On the other hand, these observations were accompanied by cytoskeleton reorganization and increased electrical signal generated by K+ channels [79]. This suggests a potential role for endocannabinoids in some pathologic conditions characterized by enhanced endometrial cell invasiveness.

Decidualization process definitively contributes to the complex maternal-fetal relationships within placental bed crucial for normal pregnancy. Taken together, there is now sufficient evidence implicating endocannabinoid elements in decidualization process. On the other hand, a disruption in endocannabinoid levels may interfere with decidual tissue remodelling and consequently with trophoblast differentiation/proliferation or invasion, ultimately impairing placental function.

The significance of COX-2 and prostaglandins for the initiation and maintenance of decidualization is well established. COX-2 is restricted to implantation sites in most species, and targeted disruption of COX-2 in mice results in multiple reproductive impairments including decidualization [80].

FAAH is responsible for the metabolism of AEA to arachidonic acid, which provides a source for prostaglandins production. Anandamide is also a direct substrate to COX-2 oxidative metabolism eventually producing prostaglandin-ethanolamides (PG-EAs). 

Some studies have recently shown that AEA is capable of modulating the production of prostaglandins. Consequently, induction of COX-2 expression may represent an underlying mechanism by which PGs may mediate eCB-dependent effects or vice versa [81–83]. In the amnion, AEA caused a significant increase in PGE2 through CB1 [81, 84]. Similarly, it was described that AEA exerts opposite effects on PGE2 and F2*α* in mice uterine explants [84]. Moreover, COX-2 derivatives mediate anandamide-inhibitory effect on nitric oxide synthase activity in the receptive uterus [85, 86]. 

Low FAAH activity and increased AEA levels are apparent in peripheral lymphocytes in women with recurrent miscarriage or poor implantation in women undergoing *in vitro* fertilization [87]. Furthermore, FAAH expression was absent in trophoblasts cells of women who miscarried [88]. Thus, when FAAH activity is absent or low, AEA goes through an oxidative metabolism primarily by COX-2 driving to prostamide production. The longer half-life of prostamides raises the possibility that they might act as mediators, and they are currently the target of studies to explore their potential pathophysiological effects. Endocannabinoid-induced effects were described to be mediated by prostamides in tumorigenic keratinocytes [89] and in other systems [90–92].

A latent biochemical cross-talk between the endocannabinoid and eicosanoid network is manifest. Furthermore, it is possible that aberrant endocannabinoid signalling may overwhelm eicosanoid expression compromising decidualization process and, in that way, fetoplacental development.

## 4. Endocannabinoid System during Placental Development

The placenta is a specialized pregnancy-specific structure that develops concurrently with the development of the embryo, being comprised of numerous cell types. Among them are specialized cells named trophoblasts, which are the earliest extraembryonic cells to differentiate from the mammalian embryo cells and surround the foetus throughout gestation.

Trophoblast cells are in direct contact with maternal tissues and play key roles in protecting the embryo/foetus from noxious substances, programming maternal support, and preventing maternal immune rejection. At the same time, they ensure appropriate bidirectional nutrient/waste flow required for growth and maturation of the embryo, enabling viviparous development. Thus, placentation is fundamental, creating the milieu, in which the embryo and foetus develop, assures a successful pregnancy, and even influences all the postnatal health and disease.

The balance between molecules synthesized by trophoblasts that promote invasion and inhibitors of this process, produced by decidua, controls the trophoblast invasiveness [93–95]. In turn, imbalances on either side can lead to abnormal invasion, resulting in pregnancy problems. Although the underlying mechanisms of placentation remain largely unknown, endocannabinoid signalling may play an important role in this process (Figure 1). 

Supported on experimental models indicating the deleterious action of cannabinoids in early pregnancy, some clinical studies about the effects of endocannabinoids on placentation have been published. Human first trimester placental tissues express FAAH and CB1, indicating human placenta as a target for cannabinoid action and metabolism [96, 97]. The higher levels of FAAH were observed in villous cytotrophoblasts and syncytiotrophoblasts, which correspond to the placental layers closest to the maternal blood [97], indicating that FAAH expression would be essential in the placenta during early pregnancy to protect the foetus from detrimental high levels of maternal AEA. 

Some studies have addressed the association between FAAH expression and recurrent miscarriage. One study observed that invasive trophoblasts and decidual cells expressed significantly more FAAH in placenta from women with recurrent miscarriage than in those of normal pregnancies [58]. This indicates an inadequate control of the endocannabinoid system in the uterus of women who experience recurrent miscarriages. However, a contradictory result has been observed with lower FAAH and high CB1 expression in placental samples of spontaneous miscarriage as compared to normal pregnancy [88]. Moreover, this study also revealed *nape-pld* transcripts, providing evidence for a potential endogenous synthesis of AEA by first trimester human placenta [88].

More recently, contrary to FAAH, NAPE-PLD expression was shown to be significantly higher in preeclamptic than in normal placentas, though no differences were observed in CB1 expression [98]. It was also hypothesized that AEA has an important implication in the normal function of placental tissues by modulating nitric oxide synthase (NOS) activity. In fact, it was observed that AEA modulates rat NO placental levels by two independent pathways: by stimulating NO synthesis via TRPV1 or diminishing the NOS activity via cannabinoid receptors, which depends on the production of cyclooxygenase-2 derivatives [85, 99]. Since placental villous from women with preeclampsia presented amplified NOS activity, increased AEA levels may be due to higher NAPE-PLD expression [98]. 

Also, in rodents a fully endocannabinoid system in placenta was described. The levels of both major endocannabinoids in the placenta gradually increased reaching their maximum level by the end of pregnancy. This increase was accompanied by higher expression of respective synthesizing enzymes, whereas the hydrolysing enzymes remained unchanged in placenta throughout pregnancy [100]. It suggests that, since expression of hydrolysing enzymes was unaffected, the high levels of both endocannabinoids are, therefore, regulated by the synthesizing enzymes. Additionally, FAAH activity was maintained constant during placentation, whereas NAPE-PLD activity increased significantly by the end of pregnancy to support the increased AEA levels observed during labour (unpublished data).

Trophoblast cell differentiation is tightly regulated and endocannabinoid signalling appears to be relevant during such processes. It was found that ablation of CB1 receptor inhibited trophoblast cell proliferation, differentiation, and invasiveness resulting in defective placentation and fetal development. In parallel, an increase in fetal resorption rates in Cb1^−/−^ females was observed, whereas trophoblast cell proliferation and differentiation were modestly affected in Faah^−/−^ females with higher AEA levels [101, 102].

Furthermore, the exogenous cannabinoid THC and AEA have been shown to reduce BeWo trophoblast cell proliferation *in vitro* via CB2 receptor, suggesting that high AEA plasma levels may increase the risk of first trimester miscarriage [103, 104]. This may explain the detrimental effects of *cannabis* consumption, as THC crosses the placenta in a greater extent during early proliferative growth phase, and, unlike endocannabinoids, which are released on demand, THC persists for long periods within the body and thereby may impact normal gestation.

## 5. Concluding Remarks

Although the adverse effects of cannabinoids in pregnancy have been implicated for years, the exact signalling mechanisms involved remain fairly unclear. In fact, maternal marijuana use has been associated with foetal growth restrictions, spontaneous miscarriage, and cognitive deficits in infancy and adolescence.

With the discovery of cannabinoid receptors, endogenous ligands, and the enzymes involved in their metabolic pathways, a wealth of information is now available regarding the importance of cannabinoid signalling in reproduction. The AEA signalling mediated by CB1 is crucial to various female reproductive events that include embryo development, oviductal transport, and implantation. However, the involvement of endocannabinoids in the molecular dialogue governing both decidualization and placentation only recently started to be depicted.

There is now evidence that endocannabinoid system is fully expressed in maternal tissues and midgestational placentas, and the levels of its constituents fluctuate during normal gestation. Additionally, CB1 receptor stimulation is involved in the inhibition of human decidualization and in the natural remodelling process occurring during this period. Moreover, endocannabinoid signalling was shown to compromise placentation through disturbing trophoblast proliferation and differentiation. CB1 knock-out mice also revealed a deficient trophoblast invasion with consequences to placentation and successful pregnancy.

There is growing evidence supporting the involvement of the endocannabinoid system in decidualization and placentation along with a possible association between polymorphism genotypes of CB1 gene and ectopic pregnancy.

AEA or 2-AG, in higher levels, represents a deleterious factor during this complex process, and a similar mechanism for exocannabinoids may occur during *cannabis* consumption in pregnancy.

This observation raises the question as to whether and how potentially increased levels of these endocannabinoids would affect the process of decidualization. It is possible that sustained higher levels might generate an imbalance in CB1 stimulation that might be responsible for an exacerbated cell death of decidual cells impairing normal placentation. On the other hand and contrary to endocannabinoids, which are synthesized “on demand” and quickly hydrolysed, THC persists for longer periods in the human body and, in that way, can interfere with normal endocannabinoid balance, either through direct stimulation of CB1 receptor and/or indirectly interfering in endocannabinoid metabolism. Thus, exogenous cannabinoid exposure may overwhelm this local protection mechanism and interfere with stromal/decidual cells, trophoblast differentiation/proliferation, and interstitial/endovascular invasion impairing placental function, which may result in intrauterine retardation and low birth weight, some of the adverse effects of *cannabis* consumption during pregnancy.
